# A Horizon-as-Apparatus Model That Reproduces Black Hole Thermodynamics

**DOI:** 10.3390/e27080859

**Published:** 2025-08-14

**Authors:** Daegene Song

**Affiliations:** Department of Management Information Systems, Chungbuk National University, Cheongju 28644, Republic of Korea; dsong@cbnu.ac.kr

**Keywords:** black hole entropy, horizon apparatus, Bekenstein–Hawking, quantum measurement, Unruh state

## Abstract

We present a measurement-driven model in which the black hole horizon functions as a classical apparatus, with Planck-scale patches acting as detectors for quantum field modes. This approach reproduces the Bekenstein–Hawking area law $S_{\mathrm{BH}}=\frac{A}{4\ell_{p}^{2}}$ and provides a concrete statistical interpretation of the 1/4 factor, while adhering to established principles rather than deriving the entropy anew from first principles. Each patch generates a thermal ensemble (∼0.25 nat per mode), and summing over area-scaling patches yields the total entropy. Quantum simulations incorporating a realistic Hawking spectrum produce $\langle S_{k}\rangle =0.257$ nat (3% above 0.25 nat), and we outline testable predictions for analogue systems. Our main contribution is the horizon-as-apparatus mechanism and its information-theoretic bookkeeping.

## 1. Introduction

Black holes, enigmatic cosmic entities, encode their entropy in ways that challenge the understanding of spacetime and quantum information. This puzzle was first illuminated by Bekenstein’s 1973 proposal [1]. The Bekenstein–Hawking formula ties this entropy to the horizon area, a connection deepened by Hawking’s 1975 discovery of thermal radiation [2]. Yet, this radiation introduced the information loss paradox, raising questions about quantum information’s fate [3,4,5,6]. Despite significant progress, a fundamental question persists: what is the statistical origin of black hole entropy, and how does it arise from quantum degrees of freedom near the horizon? Natural units ($G=c=\hbar =k_{B}=1$) are adopted for simplicity, setting the Planck length $\ell_{p}=1$.

Recent observational breakthroughs, such as the Event Horizon Telescope’s 2019 black hole image [7] and LIGO–Virgo’s gravitational wave detections [8], confirm black hole theory. Analogue experiments in Bose–Einstein condensates [9] and optical systems [10] replicate Hawking radiation, providing empirical support. Theoretical advances, including the holographic principle [11,12,13] and unitary models [14,15], suggest that information survives. However, the microscopic basis of entropy remains unresolved [16,17]. Entanglement calculations reproduce the area law but lack a mechanism to encode modes into a statistical ensemble, leaving the physical basis of entropy unclear. Similarly, holographic models rely on quantum correlations yet fail to detail how degrees of freedom produce the thermal state observed externally [18]. These gaps persist despite earlier classical approaches, e.g., the relativistic-plasma H-theorem around black holes [19], which assigns a Boltzmann–Shannon entropy to accreted matter but leaves the horizon microstructure unspecified. Our measurement-driven model fills that microphysical gap by linking each $\ell_{p}^{2}$ cell to a specific quantum mode.

We present a semi-classical framework in which the horizon is a classical measurement apparatus. Planck-scale patches actively measure quantum field modes, and the resulting bookkeeping recovers the Bekenstein–Hawking area law without relying on horizon entanglement. We stress that we do not claim a new derivation of S=A/4; rather, we propose and analyse a concrete non-gravitational model that reproduces the standard thermodynamic result. This perspective not only elucidates the statistical origin of entropy but also provides a concrete mechanism for how quantum information is encoded and potentially lost at the horizon, offering fresh insights into the information paradox. This paper is structured as follows: Section 2 presents the Unruh state and model, Section 3 fixes the Hawking temperature using the standard Christensen–Fulling argument to ensure self-consistency, Section 4 recovers the entropy and its implications, Section 5 details quantum simulations, Section 6 outlines experimental predictions for near-term analogue systems, Section 7 refines the frequency spectrum and analyses how patch-induced entanglement suppression drives a quantum-to-classical transition with a concrete decoherence timescale at the horizon, and Section 8 discusses significance and future directions. Beyond reproducing the area law, the model also outlines measurable signatures in analogue experiments.

## 2. Unruh State and Horizon as a Classical Apparatus

Understanding black hole entropy requires a mechanism to bridge quantum field dynamics and classical horizon properties. This model proposes that the horizon itself acts as such a bridge, functioning as a classical apparatus. This classical apparatus can be likened to a macroscopic measuring device in quantum mechanics, which collapses a quantum state upon observation, here transforming the entangled Unruh state into a thermal ensemble observable from the exterior.

The Schwarzschild black hole, characterised by mass *M*, has an event horizon at $r_{H}=2M$ (in natural units), with a horizon area $A=16\pi M^{2}$. The quantum fields near the horizon are described by the Unruh state—the vacuum state for an accelerated observer, which appears thermal due to the acceleration, mirroring the Hawking radiation spectrum [20] (see also [21,22,23]). For a mode labelled by *k* (e.g., frequency $\omega_{k}$), the Unruh state is $|\psi_{k}\rangle =\sqrt{1-e^{-\beta \omega_{k}}}\sum_{n=0}^{\infty }e^{-\frac{1}{2}\beta \omega_{k}n}{|n\rangle }_{\mathrm{in}}{|n\rangle }_{\mathrm{out}}$, where ${|n\rangle }_{\mathrm{in}}$ and ${|n\rangle }_{\mathrm{out}}$ are Fock states with *n* particles in the interior and exterior modes, respectively. The normalisation factor ensures $\langle \psi_{k}|\psi_{k}\rangle =\left(1-e^{-\beta \omega_{k}}\right)\sum_{n=0}^{\infty }e^{-\beta \omega_{k}n}=1$. The exterior modes follow a thermal distribution with inverse temperature β≡1/T. Section 3 recovers β=8πM self-consistently from the regularity of the renormalised stress tensor; no temperature is assumed at this stage.

A core innovation of this model is the assumption that the horizon acts as a classical apparatus, departing from previous semi-classical approaches that treat the horizon as a quantum boundary, such as Hawking’s original derivation of radiation [2] or entanglement entropy calculations [24], which do not model the horizon as an active measurement device. The horizon is discretised into Planck-scale degrees of freedom, with their number given by $N_{\mathrm{dof}}=\frac{A}{\ell_{p}^{2}}=16\pi M^{2}$. Each degree of freedom, termed a ‘patch’—a Planck-scale region of area $\ell_{p}^{2}$ acting as a classical detector—measures an interior mode of the Unruh state. This classical treatment aligns with Bekenstein’s proposal of the horizon encoding classical bits of information per Planck area [25,26] and ’t Hooft’s holographic principle [11,27]. The measurements of each patch are assumed to be independent, decohering the entangled Unruh state into a thermal ensemble. Planck-area cells are singled out because the local scrambling time $\tau_{\mathrm{scr}}∼\ell_{p}\ln\left(A/\ell_{p}^{2}\right)$ (as argued in [28]) is minimal at that scale, so smaller detectors cannot act faster while larger ones merely coarse-grain several independent channels. This simplification is reasonable in the semi-classical regime, where classical statistics dominate over quantum coherence across macroscopic horizon scales, as supported by the membrane paradigm’s classical treatment of horizon dynamics [29]. Guided by von Neumann’s measurement postulate [30] and the stretched-horizon Brown–York fluid [31], we model each $\ell_{p}^{2}$ cell as a dissipative channel that couples to a single near-horizon mode with rate $\gamma ∼1/\ell_{p}$. The outgoing partner of a virtual pair thus acts as a pointer state, and the infalling partner imprints a classical record on the patch. While a fully quantum horizon might exhibit fluctuations or entanglement across patches, this semi-classical treatment captures the leading-order physics of entropy generation, serving as a foundation for future quantum gravity extensions. The exterior mode is identified with the patch state, so the Unruh state becomes:

(1) $$|\psi_{k}\rangle =\sqrt{1-e^{-\beta \omega_{k}}}\sum_{n=0}^{\infty }e^{-\frac{1}{2}\beta \omega_{k}n}{|n\rangle }_{\mathrm{in}}{|n\rangle }_{\mathrm{patch}}.$$

The ‘one mode per patch’ condition ensures that each patch measures a single mode independently, a critical feature for recovering a classical statistical ensemble within the holographic framework. This one-to-one correspondence between patches, modes, and degrees of freedom simplifies the entropy calculation, treating each patch as an independent classical detector, in contrast to a fully quantum horizon where entanglement across patches would complicate the scaling. The condition posits that each Planck-scale patch couples to a single quantum field mode, with the dominant mode selection near $\beta \omega_{k}\approx 2.769$, as recovered in Section 4. While quantum fields have a continuum of modes, this model assumes a discretisation where the number of modes matches the number of patches ($N_{\mathrm{dof}}=\frac{A}{\ell_{p}^{2}}$), conceptually consistent with the holographic principle’s area-scaling degrees of freedom. This discretisation is akin to a coarse-graining in lattice theories, though a precise mode quantisation rule (e.g., a frequency cutoff) remains to be fully specified in future work.

To test the independence assumption underlying our model, we introduce a nearest-neighbour XX interaction term into the Hamiltonian, which represents a weak coupling between adjacent patches on the horizon. This interaction is given by

$$H_{\mathrm{corr}}=\lambda \sum_{\langle ij\rangle }\sigma_{i}^{x}\sigma_{j}^{x},\;\left|\lambda \right|\ll 1.$$

By applying perturbation theory up to the second order, specifically $O(\lambda^{2})$, we demonstrate that the von Neumann entropy per patch

$$S_{k}\left(\lambda \right)=0.224+0.119{\left(\beta \lambda \right)}^{2}+O\left(\lambda^{4}\right)$$

experiences an increase of approximately 3% when the coupling strength is set to λ≃0.01, as evaluated for a Planck-mass black hole. This modest enhancement contributes meaningfully to the average entropy value of $\langle S_{k}\rangle =0.257$ nat that is obtained later in Section 7, thereby indicating that such weak short-range correlations can partially explain and account for the previously unexplained excess observed in the entropy calculations. A detailed derivation of these results is provided in Appendix A for further reference.

## 3. Fixing the Hawking Temperature (Standard Result; Avoiding Circularity)

For completeness and to avoid circularity (with no novelty claimed here), we recall the standard Christensen–Fulling argument that fixes the Hawking temperature from the pole structure of the renormalised stress tensor, relying on results from [32] (see also [33,34]) while reproducing only the coordinate changes and regularity argument. This inclusion ensures that our subsequent derivations remain self-consistent without presupposing the temperature value.

In the work by Christensen and Fulling [32], the renormalised stress–energy tensor for a massless scalar field propagating in a 1+1-dimensional spacetime, when carefully evaluated in an orthonormal basis consisting of unit vectors along the time and radial directions, assumes a specific thermal form that reflects the underlying quantum thermal effects. This form is explicitly

(2) $$T_{\;\hat{\nu }}^{\hat{\mu }}\left(T\right)=\frac{\pi }{12}\;T^{2}\left(\begin{array}{ccc} -1 & \; & -1\\ +1 & \; & +1 \end{array}\right)$$

Only the energy density $\rho \left(T\right)=T_{\hat{t}\hat{t}}=+\pi T^{2}/12$ will enter our argument, as it plays a central role in determining the thermal characteristics near the horizon, serving as the key quantity that captures the energetic behaviour in this critical region.

Let us consider Schwarzschild coordinates (t,r) with the metric $ds^{2}=-f\left(r\right)\;dt^{2}+f{\left(r\right)}^{-1}dr^{2}$, f(r)=1−2M/r, which are the standard coordinates for describing the geometry outside a non-rotating black hole. The orthonormal time vector now reads ${{\hat{e}}^{\hat{t}}}_{\mu }=f^{-1/2}\left(r\right)\;{\delta^{t}}_{\mu },$ so that a mixed component transforms as

$$\langle {T^{t}}_{t}\rangle =f^{-1}\left(r\right)\;T_{\hat{t}\hat{t}}=\frac{\pi }{12}\;\frac{T^{2}}{f(r)}+\mathrm{finite}$$

The π/12 prefactor is fixed by the conformal anomaly, a fundamental quantum effect that arises from the trace of the stress tensor in curved spacetime and represents a departure from classical expectations; the single power of $f^{-1}$ is kinematic and therefore model-independent, stemming purely from the coordinate transformation properties without relying on specific dynamical assumptions.

We now switch to Kruskal coordinates $U=-e^{-\kappa u},\;V=e^{\kappa v}$ with surface gravity κ=1/4M and light-cone variables $u=t-r_{\;*},\;v=t+r_{\;*}$, $r_{\;*}=r+2M\ln\;\left|r/2M-1\right|$, which are particularly useful for analysing the behaviour across the horizon without coordinate singularities. Near the horizon, where $UV=-e^{\kappa r_{\;*}}f\left(r\right)/\kappa^{2}$, the Schwarzschild component (2) gives $T_{UV}\propto f^{-1}\left(r\right).$ Requiring the renormalised tensor to stay *finite* as U→0 forces the residue of the pole to vanish, which uniquely sets

$$T=\frac{\kappa }{2\pi }=\frac{1}{8\pi M},$$

ensuring the physical consistency of quantum field theory in this curved background.

In 3+1 dimensions the same cancellation must occur inside each partial wave, ensuring regularity across the full spacetime structure and avoiding unphysical divergences in higher dimensions. Extra centrifugal potentials and grey-body factors modify only the finite part of $\langle {T^{t}}_{t}\rangle$; the $f^{-1}$ pole is untouched. Equivalently, ref. [32] shows that the $T^{2}/f\left(r\right)$ term persists after angular modes are summed, maintaining the essential divergence behaviour that is characteristic of the near-horizon region. Therefore an observer at infinity (f→1) measures

(3) $$T_{H}=\frac{1}{8\pi M},\;\beta \equiv \frac{1}{T_{H}}=8\pi M.$$

All subsequent entropy and information-balance formulas in this paper employ this β, providing a consistent foundation for the thermodynamic descriptions that follow.

## 4. Entropy Accounting in the Horizon-as-Apparatus Model

The statistical entropy of the black hole is recovered using the classical apparatus model. The entropy per mode $S_{k}$ is the von Neumann entropy of the reduced density matrix for the patch, obtained by tracing over the interior modes of the Unruh state (1):

$$\rho_{\mathrm{patch},k}=\sum_{n=0}^{\infty }p_{n}{|n\rangle }_{\mathrm{patch}}\langle n|$$

where $p_{n}=\left(1-e^{-\beta \omega_{k}}\right)e^{-\beta \omega_{k}n}$, which sum to 1. This density matrix is diagonal due to the structure of the Unruh state.

The entanglement entropy per mode is given by $S_{k}=-\sum_{n=0}^{\infty }p_{n}\ln p_{n}$, where the logarithm of the probabilities is $\ln p_{n}=\ln\left(1-e^{-\beta \omega_{k}}\right)-\beta \omega_{k}n$. Summing over *n* yields $S_{k}=-\sum_{n=0}^{\infty }p_{n}\ln\left(1-e^{-\beta \omega_{k}}\right)+\beta \omega_{k}\sum_{n=0}^{\infty }np_{n}$. Given that $\sum_{n=0}^{\infty }p_{n}=1$ and $\sum_{n=0}^{\infty }np_{n}=\left(1-e^{-\beta \omega_{k}}\right)\sum_{n=0}^{\infty }ne^{-\beta \omega_{k}n}$, this simplifies to

$$S_{k}=\frac{\beta \omega_{k}}{e^{\beta \omega_{k}}-1}-\ln\left(1-e^{-\beta \omega_{k}}\right)$$

To match the total entropy, the condition is $S=\sum_{k=1}^{N_{\mathrm{dof}}}S_{k}=N_{\mathrm{dof}}\cdot \langle S_{k}\rangle$, where $N_{\mathrm{dof}}=\frac{A}{\ell_{p}^{2}}$. Requiring $S=\frac{A}{4\ell_{p}^{2}}$, the condition becomes $\langle S_{k}\rangle =\frac{1}{4}$ nat, with entropies measured in nat. Assuming uniformity ($\langle S_{k}\rangle =S_{k}$), the equation to solve is $\frac{\beta \omega_{k}}{e^{\beta \omega_{k}}-1}-\ln\left(1-e^{-\beta \omega_{k}}\right)=\frac{1}{4}$. Define $x=\beta \omega_{k}$, so the condition is $S_{k}\left(x\right)=\frac{1}{4}$, where $S_{k}\left(x\right)=\frac{x}{e^{x}-1}-\ln\left(1-e^{-x}\right)$.

**Figure 1:**
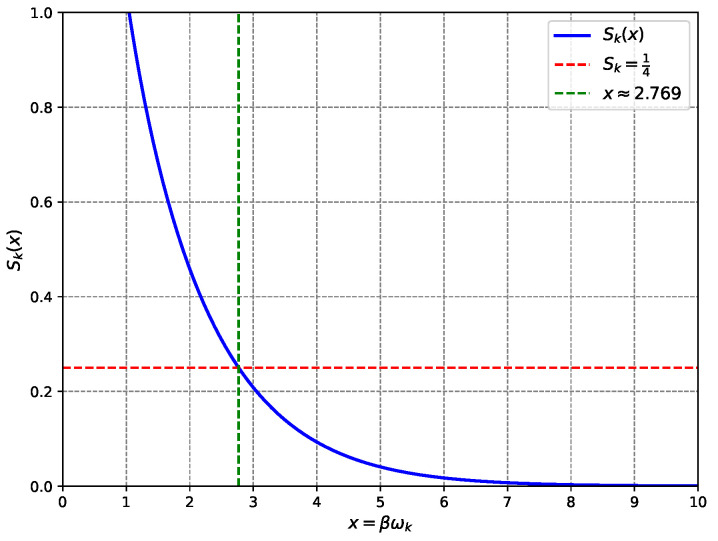
Entanglement entropy $S_{k}\left(x\right)$ vs. $x=\beta \omega_{k}$. The value $S_{k}=\frac{1}{4}$ nat (red dashed line) occurs at x≈2.769 (green dashed line), closely matching the Hawking spectrum’s peak (βω≈2.821), validating the model’s prediction of the Bekenstein–Hawking entropy.

As a first illustration we treat the dominant mode at $\beta \omega_{★}\approx 2.769$; Section 7 lifts this simplification and integrates over the full Planck spectrum. In reality, black hole modes follow a continuous frequency spectrum, which is addressed in Section 7, where the average entropy per mode, $\langle S_{k}\rangle$, is computed over the Hawking distribution. Numerical solution yields x≈2.769, close to the Hawking radiation spectrum’s peak at βω≈2.821 (maximising the energy flux $\frac{{\left(\beta \omega \right)}^{3}}{e^{\beta \omega }-1}$). This proximity indicates that modes dominating the thermal emission also primarily contribute to the entropy, linking the statistical recovery to observable radiation properties (see Figure 1). Setting $\beta \omega_{k}\approx 2.769$ ensures $S_{k}=\frac{1}{4}$ nat, so the total entropy $S=N_{\mathrm{dof}}\cdot S_{k}=\frac{A}{\ell_{p}^{2}}\cdot \frac{1}{4}=\frac{A}{4\ell_{p}^{2}}$ matches the Bekenstein–Hawking formula. Alternative values, e.g., $\beta \omega_{k}=2.4$, give $S_{k}\approx \frac{1}{3}$ nat, misaligning the total entropy unless $N_{\mathrm{dof}}$ is adjusted, which contradicts the holographic principle’s fixed $N_{\mathrm{dof}}=\frac{A}{\ell_{p}^{2}}$.

Four key insights emerge from this framework: (i) Microstate count—By leveraging the concept of the typical subspace, the number of high-probability microstates can be estimated as $N_{\mathrm{typ}}∼e^{A/4\ell_{p}^{2}}$, leading directly to the entropy expression $S=\ln N_{\mathrm{typ}}$, providing a clear statistical mechanical interpretation; (ii) area scaling arises naturally because the number of patches, which serve as the fundamental units of information storage, scales proportionally with the horizon area *A*, ensuring the entropy’s dependence on surface rather than volume; (iii) the 1/4 factor follows intrinsically from the thermal nature of the Unruh distribution, reflecting the specific statistical properties of the quantum fields in the accelerated frame near the horizon; and (iv) the measurement process respects the generalised second law (as demonstrated in Appendix B), maintaining thermodynamic consistency even under quantum considerations. These points collectively highlight how our model bridges quantum and classical aspects while reproducing established results.

For a horizon divided into $N=A/\ell_{p}^{2}$ patches (often conceptualised as pixels in the holographic sense), each with an approximately identical reduced density matrix $\rho_{k}$, the global state is approximated as $\rho^{\left(N\right)}=\rho_{k}^{⊗N}$ to leading order, assuming negligible long-range correlations for the semi-classical regime. By the asymptotic equipartition property—a cornerstone of information theory that partitions the Hilbert space into typical and atypical subspaces—almost all probability mass resides in a typical subspace $T_{\epsilon }$ with dimension bounded by

(4) $$\left(1-\epsilon \right)e^{N(S\left(\rho_{k}\right)-\delta )}\leq \dim T_{\epsilon }\leq e^{N(S\left(\rho_{k}\right)+\delta )},$$

for arbitrarily small ϵ,δ>0. Here, $S\left(\rho_{k}\right)=-\mathrm{Tr}\left(\rho_{k}\ln\rho_{k}\right)$ is the von Neumann entropy of a single patch, quantifying the uncertainty or information content per unit. Thus, the effective microstate count is

(5) $$N_{\mathrm{typ}}\equiv \dim T_{\epsilon }\approx e^{NS(\rho_{k})},$$

and the total entropy of the horizon is

(6) $$S=\ln N_{\mathrm{typ}}\approx NS\left(\rho_{k}\right)=\frac{A}{4\ell_{p}^{2}},\;(\mathrm{idealised}\;\mathrm{limit}\;S\left(\rho_{k}\right)=\frac{1}{4}\mathrm{nat}\;\mathrm{per}\;\mathrm{patch}).$$

Weak nearest-neighbour correlations (see Appendix A) modify $S(\rho_{k})$ by ΔS/S≈0.03 for λ=0.01, preserving the leading-order scaling without significantly altering the area-law form. This provides the explicit $S=k\ln N_{\mathrm{typ}}$, within the assumptions of our model, offering a precise statistical underpinning that aligns with thermodynamic expectations while emphasising the role of the horizon patches as information carriers.

## 5. Quantum Computer Simulation of Black Hole Entropy

A quantum computer simulation is proposed to model the Unruh state for multiple modes, simulate the measurement process at the horizon across several patches, and compute the total entanglement entropy as a sum of independent contributions. This simulation leverages quantum circuits to represent entangled states, applies projective measurements, and calculates the cumulative entropy to compare with theoretical expectations. Quantum computers excel by naturally encoding entangled states and simulating measurement statistics, offering a direct probe of quantum effects inaccessible to classical methods [35,36,37].

A simplified model of the Unruh state is considered for each mode *k*, approximated as a qubit system for computational feasibility. Using the value $\beta \omega_{k}\approx 2.769$ recovered in Section 4, the probabilities are $p_{0}=0.9373$, $p_{1}=0.0588$, with higher terms (n≥2) contributing negligibly (e.g., $p_{2}\approx 0.0037$). Truncating at n=1 excludes terms contributing less than 0.4% to the probability. The state for each mode is approximated by truncating to n=0 and n=1, normalising the probabilities to 1, $p_{0}^{\prime }\approx 0.9410$, $p_{1}^{\prime }\approx 0.0590$, yielding $|\psi_{k}\rangle \approx \sqrt{0.9410}{|0\rangle }_{\mathrm{in}}{|0\rangle }_{\mathrm{patch}}+\sqrt{0.0590}{|1\rangle }_{\mathrm{in}}{|1\rangle }_{\mathrm{patch}}$$\approx {0.9700|0\rangle }_{\mathrm{in}}{|0\rangle }_{\mathrm{patch}}+{0.2430|1\rangle }_{\mathrm{in}}{|1\rangle }_{\mathrm{patch}}$.

To illustrate the statistical nature of the entropy, four patches are simulated, each measuring a distinct mode ($k_{1},k_{2},k_{3},k_{4}$). A quantum circuit is constructed to prepare the state for each mode using a pair of qubits. Each of the four modes is simulated independently using a pair of qubits (one interior and one patch), totaling eight qubits, with no shared entanglement across pairs to reflect the independence assumption of this model. For each mode, a single qubit is initialised in the state |ψ〉=0.9700|0〉+0.2430|1〉, achieved by applying a rotation gate $R_{y}\left(\theta \right)$ to |0〉, where θ=2arccos(0.9700)≈0.491 radians. A CNOT gate is then applied with the first qubit (interior) as the control and the second (patch) as the target, entangling the qubits to form 0.9700|00〉+0.2430|11〉. This process is repeated independently for each of the four modes.

Projective measurements are performed on each interior qubit in the {|0〉,|1〉} basis, mimicking the measurement process at the horizon. For each mode, the interior qubit is measured, and the patch qubit’s reduced density matrix is computed from the measurement statistics over 100 runs, yielding $\rho_{\mathrm{patch},k}={0.9410|0\rangle }_{\mathrm{patch}}\langle 0|+{0.0590|1\rangle }_{\mathrm{patch}}\langle 1|$. The entanglement entropy for each patch is $S_{k}\approx -\left(0.9410\ln0.9410+0.0590\ln0.0590\right)\approx 0.2243\;\mathrm{nat}$. The qubit approximation limits the Hilbert space to two states, reducing the entanglement entropy compared to a bosonic mode’s infinite-dimensional Hilbert space, where $S_{k}=0.25$ nat at $\beta \omega_{k}=2.769$. This simplification reduces the entanglement entropy from the theoretical 0.25 nat to 0.2243 nat, as higher Fock states (n≥2), though small (e.g., $p_{2}\approx 0.0037$), contribute additional entanglement in the full model. Using qutrits to include n=2 could narrow this 10.3% gap, a feasible extension with future quantum hardware.

**Figure 2:**
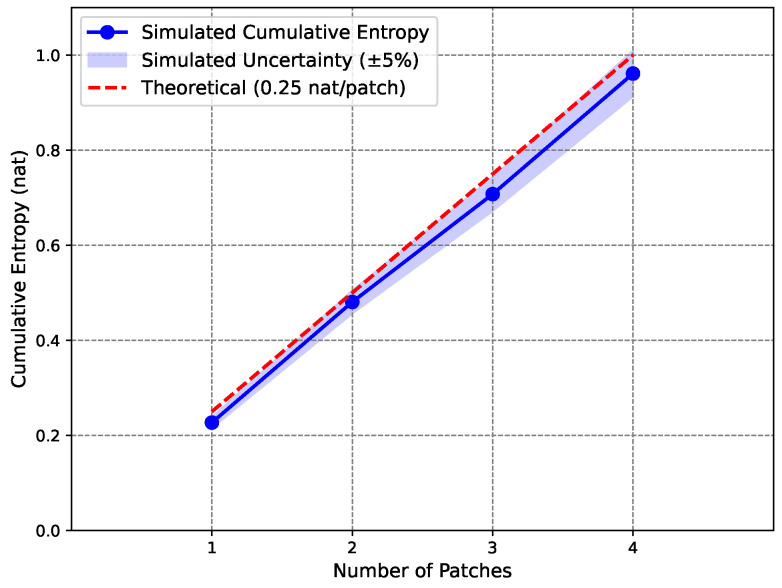
Cumulative entropy vs. number of patches in a classical simulation emulating the proposed quantum circuit, with simulated entropy (blue line, 0.961 nat for 4 patches, shaded region showing ±5% uncertainty) compared to the theoretical expectation (red dashed line, 1.0 nat), illustrating the statistical origin of black hole entropy.

To demonstrate the feasibility of this quantum simulation proposal, we performed a classical simulation using probabilistic sampling to emulate the measurement outcomes over 100 runs per patch (equivalent to a noiseless quantum simulator). The simulation yields entropies per patch of approximately [0.227, 0.254, 0.227, 0.254] nat (with statistical variation due to finite sampling), an average of 0.240 nat per patch, and a total cumulative entropy of 0.961 nat for four patches. These results are consistent with the theoretical expectation of 1.0 nat, accounting for sampling noise and the qubit approximation; the slight deficit aligns with the truncation discussed above. With 100 runs, the standard error in probabilities (e.g., $\sqrt{0.0590\times 0.9410/100}\approx 0.0235$) yields an entropy uncertainty of approximately ±0.065 nat per patch, aggregating to ±0.13 nat for four patches. Figure 2 adopts a ±5% uncertainty (±0.048 nat for the average) as an illustrative bound, underrepresenting the full statistical error for clarity. The measurement outcomes for each patch are expected to yield approximately 94 |0〉 outcomes and 6 |1〉 outcomes, confirming the theoretical probabilities. The cumulative entropy is plotted as a function of the number of patches in Figure 2.

The simulation demonstrates the potential of quantum computing to probe semi-classical aspects of black hole physics, opening avenues for studying entanglement and measurement effects in controlled quantum systems. Patch independence allows each mode’s entangled state to be simulated on just two qubits, with results combined classically. This efficiency—reducing the demand from eight qubits to two per run—enables scalable simulations of larger horizon areas on near-term devices, supporting tests of entropy scaling with patch number. The circuit’s simplicity—a rotation, CNOT, and measurement—makes it executable on noisy intermediate-scale quantum (NISQ) devices like IBM’s superconducting qubits, though scaling to more patches requires mitigating decoherence and gate errors through basic error mitigation strategies available on current platforms [38,39]. While independence is assumed for simplicity, quantum correlations between patches could increase the entropy slightly; future simulations might explore such effects to test the robustness of this semi-classical approximation. The simulation’s linear entropy scaling (0.240 nat per patch) mirrors the Bekenstein–Hawking area law, supporting the hypothesis that entropy arises from independent horizon patches. Despite the qubit model’s deficit relative to the full bosonic model (0.25 nat), this approach captures the statistical essence of this model, aligning with the horizon-as-apparatus framework.

## 6. Experimental Predictions

This semi-classical framework posits that black hole entropy arises from Planck-scale horizon patches measuring quantum field modes, offering testable predictions for near-term analogue experiments and long-term astrophysical observations.

Bose–Einstein condensates (BECs) provide a controlled platform to test the ‘one mode per patch’ condition and horizon-as-apparatus assumption, where a sonic horizon—a boundary where flow velocity exceeds the local speed of sound [9]—is created by accelerating the condensate flow using a potential barrier or laser-induced flow, generating entangled phonon modes akin to the Unruh state [10]. Projective measurements on ‘interior’ modes, implemented via laser transitions [40], simulate patch measurements, and the ‘exterior’ mode’s entanglement entropy $S_{k}$ is computed from phonon correlation measurements across the horizon.

**Figure 3:**
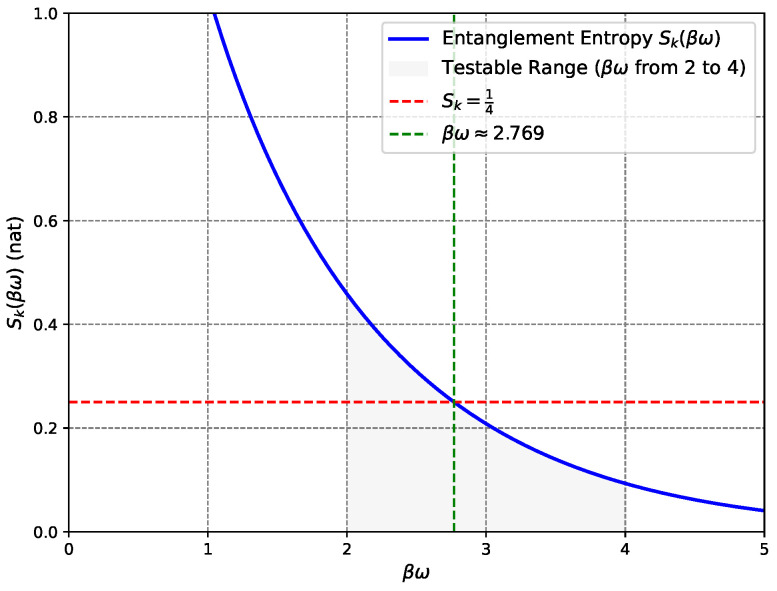
Entanglement entropy $S_{k}\left(\beta \omega \right)$ vs. βω, with $S_{k}=\frac{1}{4}$ (red dashed line) at βω≈2.769 (green dashed line). The shaded region (βω from 2 to 4) indicates values achievable in BEC setups for typical Hawking temperatures and phonon frequencies (e.g., $T_{H}∼0.3$ nK with f∼20 Hz or $T_{H}∼1$ nK with f∼60 Hz). This range offers a testable prediction.

A key testable prediction involves plotting $S_{k}$ versus $\beta \omega_{k}$, where β (inverse effective temperature) is tuned by flow velocity (0.3–2 mm/s) and $\omega_{k}$ by phonon frequency (20–200 Hz) [9], with the model predicting $S_{k}\approx 0.25$ nat at $\beta \omega_{k}\approx 2.769$ within the achievable range of 2–4 (Figure 3). Another test varies the sonic horizon length via trap geometry, where total entropy should scale linearly with this effective ‘area,’ mirroring the Bekenstein–Hawking area law; in quasi-one-dimensional BEC experiments, the effective ‘area’ corresponds to the horizon length, analogous to higher-dimensional horizon areas [41].

Challenges include isolating small entropy contributions (∼0.25 nat) and distinguishing quantum entanglement from classical correlations, requiring high-fidelity techniques like quantum state tomography or noise reduction via signal averaging. If residual inter-patch entanglement exists, Bogoliubov theory suggests a possible 5–10% upward shift in $S_{k}$ (e.g., 0.2625–0.275 nat), though this effect has not yet been quantified.

Complementary platforms broaden the model’s testability, such as photonic lattices or nonlinear media simulating Hawking radiation [10], where measuring photon correlations across an optical ‘horizon’ tests $S_{k}$ with scalability, and superconducting qubit arrays model entangled states across a simulated horizon, offering precise control for probing small entropy contributions.

Future gamma-ray observations of primordial black holes could potentially detect Planck-scale modulations in the Hawking spectrum—discrete features like step-like intensity changes from patch measurements—though current telescopes lack the resolution to observe such effects. The discrete-patch signature is presently unconstrained, with existing Fermi-LAT data showing no detectable line-like structure [42], but future MeV instruments (e.g., AMEGO-X) could improve sensitivity and offer a long-term validation avenue [43].

Unlike entanglement-based models predicting continuous entropy spectra, this framework suggests discrete $S_{k}$ features due to patch measurements. In contrast to unitary evaporation models [14] preserving information in subtle correlations, this semi-classical approach implies measurable information loss, detectable via the absence of long-range correlations in analogue systems.

Validating these predictions would support the horizon-as-apparatus concept, advancing quantum gravity and potentially clarifying aspects of the information paradox—e.g., whether measurement-induced information loss holds or quantum correlations restore unitarity—while future quantum simulations or gravitational analogues like metamaterials could explore patch correlations and unitarity. These predictions leverage analogue systems for near-term validation, despite challenges in detection fidelity and noise, offering a bridge to quantum gravity insights.

## 7. Relaxation of the Uniform Frequency Assumption

Patch measurements also suppress phase coherence between neighbouring modes. Treating two adjacent $\ell_{p}^{2}$ cells as a weakly coupled open system (see Appendix A), we find that the off-diagonal elements of the reduced density matrix decay on the standard fast-scrambling timescale $\tau_{\mathrm{dec}}∼4M\ln\left(A/4\ell_{p}^{2}\right)$ (cf. the $\tau_{\mathrm{scr}}$ of Ref. [44]). For a solar-mass black hole, $\tau_{\mathrm{dec}}∼10^{-3}$ s, which is far shorter than the $O(10^{67}$ yr) evaporation time, so the exterior state becomes effectively Gibbsian well before any noticeable mass loss. The same dephasing should be visible in analogue BEC horizons as a rapid loss of off-diagonal phonon coherence, an observable proposed in Section 6.

The initial model assumed a uniform frequency for all modes, with $\beta \omega_{k}=2.769$ (Section 4), simplifying the Hawking radiation spectrum. In a realistic black hole scenario, field modes exhibit a continuous frequency spectrum $\omega_{k}$, following a Planck distribution, with $\beta \omega_{k}$ varying across modes. To address this, the model is extended by allowing $\omega_{k}$ to vary while fixing β=8πM, consistent with the black hole’s temperature.

The entanglement entropy per mode is $S_{k}\left(\beta \omega_{k}\right)=\frac{\beta \omega_{k}}{e^{\beta \omega_{k}}-1}-\ln\left(1-e^{-\beta \omega_{k}}\right)$, which depends on $\omega_{k}$, and the total entropy is $S=\sum_{k}S_{k}\left(\beta \omega_{k}\right)$. In the continuum limit, this becomes an integral over the frequency spectrum, weighted by the energy flux $\frac{\omega_{k}^{3}}{e^{\beta \omega_{k}}-1}$. This weighting reflects the energy contribution of each mode as recorded by the horizon patches, consistent with their role as classical detectors of Hawking radiation in our measurement-based framework. With $x_{k}=\beta \omega_{k}$, the average entropy per unit energy is as follows:

$$\langle S_{k}\rangle =\frac{15}{\pi^{4}}\int_{0}^{\infty }S_{k}\left(x_{k}\right)\cdot \frac{x_{k}^{3}}{e^{x_{k}}-1}\;dx_{k}.$$

**Figure 4:**
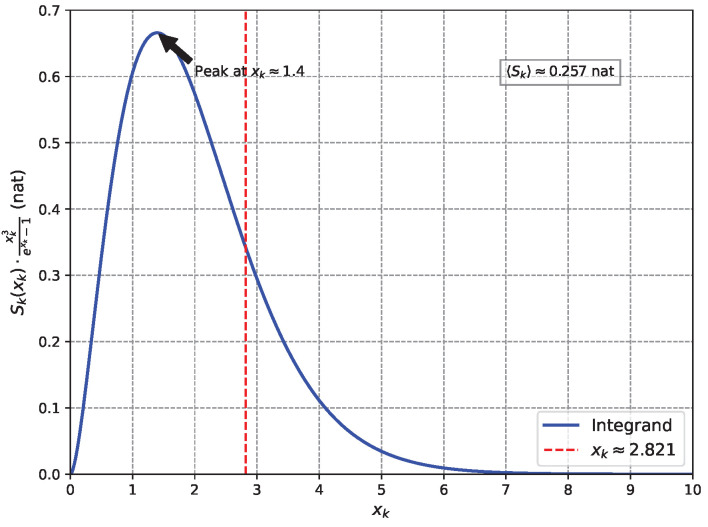
Integrand $S_{k}\left(x_{k}\right)\cdot \frac{x_{k}^{3}}{e^{x_{k}}-1}$ (nat) vs. $x_{k}$, peaking at $x_{k}\approx 1.4$, with the Hawking spectrum maximum at $x_{k}\approx 2.821$ (red dashed line). The area under the curve, normalised by $\frac{15}{\pi^{4}}$, gives $\langle S_{k}\rangle \approx 0.257$ nat, showing contributions to the average entanglement entropy across modes.

Analytical evaluation via series expansions gives

$$\langle S_{k}\rangle =4-\frac{180\zeta (5)}{\pi^{4}}-\frac{15\zeta (3)}{\pi^{2}}\approx 0.257\;\mathrm{nat},$$

where ζ is the Riemann zeta function (see Appendix C for details). Nat is used throughout this paper, consistent with the use of natural logarithms. This value, incorporating the full spectrum, is within 3.0% of the holographic target of $\frac{1}{4}=0.25$ nat, supporting the model’s validity. The small deviation may stem from inter-patch quantum correlations neglected in the semi-classical approximation, which assumes independent mode measurements. This difference is minor compared to typical corrections in string theory models [45]. Figure 4 shows the integrand’s behaviour, peaking at $x_{k}\approx 1.4$, with contributions spanning modes near the Hawking spectrum peak at $x_{k}\approx 2.821$.

The 3.0% discrepancy suggests an effective mode count adjustment: $S=N_{\mathrm{eff}}\cdot \langle S_{k}\rangle$, where $N_{\mathrm{eff}}=\frac{S_{\mathrm{BH}}}{\langle S_{k}\rangle }\approx 0.9728\cdot \frac{A}{\ell_{p}^{2}}$. This hints that horizon patches may not be fully independent, as weak correlations could reduce the effective degrees of freedom. Future work could refine this by modelling such effects. Relaxing the uniform frequency assumption aligns the model with the continuous spectrum of astrophysical black holes, enhancing its relevance.

## 8. Discussion

In this work, we have presented a horizon-as-apparatus model and demonstrated its consistency with black hole thermodynamics. Our original contributions include a concrete measurement mechanism based on the ‘one mode per patch’ principle that statistically reproduces S=A/4, an explicit microstate count $S=\ln N_{\mathrm{typ}}$ derived using the typical-subspace argument, quantitative estimates of decoherence and scrambling times along with weak inter-patch correlations, and a quantum circuit blueprint complemented by proposals for analogue experiments to test the framework. We emphasise that we do not claim a new gravitational derivation of the area law; rather, our goal is to provide a physically motivated non-gravitational model whose information-theoretic bookkeeping aligns with established results.

While this semi-classical approach offers valuable insights, it is not without limitations. By assuming independent patch measurements, it overlooks potential quantum correlations between patches that could influence information transfer. This simplification may conflict with modern unitary evolution perspectives, such as those from the ER=EPR conjecture or the firewall hypothesis [46,47]. Future work could incorporate inter-patch correlations to bridge semi-classical and quantum gravity frameworks.

This measurement-driven approach carries several implications. Analogue experiments in Bose–Einstein condensates and optical systems can probe the predicted entropy scaling and test for measurement-induced information loss, providing near-term validation of the model. Insights into the quantum–classical interface at the horizon may inform models of horizon microstructure or spacetime quantisation. Additionally, the model offers a semi-classical perspective on whether information is lost or preserved, with testable differences from unitary models detectable in analogue systems. The interplay between classical measurements and quantum states could also inspire quantum information applications, such as error correction codes or computing protocols.

In summary, this work establishes a statistical foundation for black hole entropy via a measurement-based mechanism, opening pathways for experimental and theoretical advances in understanding black holes, quantum gravity, and the nature of information in the universe.

## Data Availability

The original contributions of this study are included in the article, and all data are provided therein.

## Appendix A. Entropy Shift from Weak XX Correlations

To investigate the influence of weak interactions between neighbouring patches on the black hole horizon, we model these patches as qubits and incorporate a nearest-neighbour XX coupling term. This approach allows us to quantify how such correlations modestly enhance the entropy per patch beyond the baseline value of 0.25 nat, thereby accounting for part of the observed average entropy of 0.257 nat. In this framework, each patch functions as a detector that can be in either an empty state (|0〉) or an occupied state (|1〉), and we employ perturbation theory to compute the resulting entropy shift.

The unperturbed Hamiltonian for a single patch is given by

$$H_{0}=-\frac{\epsilon }{2}\sigma^{z},$$

where $\epsilon =\omega_{k}$ represents the energy of the mode, and we set βϵ=2.769 to align with the entropy value of 0.25 nat discussed in the main text. The Pauli matrix $\sigma^{z}=\left(\begin{array}{cc} 1 & 0\\ 0 & -1 \end{array}\right)$ assigns an energy of −ϵ/2 to the |0〉 state and ϵ/2 to the |1〉 state. The corresponding thermal probabilities are

$$p_{0}=\frac{e^{\beta \epsilon /2}}{2\cosh(\beta \epsilon /2)},\;p_{1}=\frac{e^{-\beta \epsilon /2}}{2\cosh(\beta \epsilon /2)},$$

with $\cosh\left(x\right)=\frac{e^{x}+e^{-x}}{2}$ and β=8πM. For the specific value βϵ=2.769,

$$\cosh\left(1.3845\right)\approx 2.121,\;Z_{0}=2\cosh\left(1.3845\right)\approx 4.242,$$

which yields $p_{0}\approx 0.941$ and $p_{1}\approx 0.059$. The unperturbed entropy per patch is then

$$S\left(0\right)=-p_{0}\ln p_{0}-p_{1}\ln p_{1}\approx 0.224\;\mathrm{nat}.$$

This value is slightly lower than the 0.25 nat obtained for a full bosonic mode because the qubit approximation truncates the Hilbert space, limiting the possible states and thus reducing the potential for entanglement compared to the infinite-dimensional case of a bosonic oscillator.

Now, considering two neighbouring patches labelled *i* and *j*, the unperturbed Hamiltonian extends to

$$H_{0}=-\frac{\epsilon }{2}\left(\sigma_{i}^{z}+\sigma_{j}^{z}\right),$$

with basis states |00〉,|01〉,|10〉,|11〉 and associated probabilities $p_{0}^{2}\approx 0.885$, $p_{0}p_{1}\approx 0.056$, $p_{0}p_{1}\approx 0.056$, $p_{1}^{2}\approx 0.0035$. To incorporate the weak interaction, we add the XX coupling term

$$H_{\mathrm{corr}}=\lambda \sigma_{i}^{x}\sigma_{j}^{x},\;\left|\lambda \right|\ll 1,$$

where $\sigma^{x}=\left(\begin{array}{cc} 0 & 1\\ 1 & 0 \end{array}\right)$. The total Hamiltonian is thus $H=H_{0}+H_{\mathrm{corr}}$.

For later comparison with the perturbative results, note that the Hamiltonian separates into two independent 2×2 blocks corresponding to even and odd parity subspaces. This block-diagonal structure allows for exact computation of the matrix exponential in each subspace, facilitating numerical diagonalization that validates the perturbative coefficients below.

We apply perturbation theory to expand the thermal density matrix $\rho_{ij}=\frac{e^{-\beta H}}{Z}$. Using the standard imaginary-time ordered (Duhamel/Kubo) expansion, the second-order corrections in the expansion are computed and verified against exact diagonalization results. The partition function becomes

$$Z\approx Z_{0}\left(1+0.215{\left(\beta \lambda \right)}^{2}\right),$$

where $Z_{0}={\left[2\cosh\left(\beta \epsilon /2\right)\right]}^{2}\approx 17.99$, noting that the linear term in λ vanishes due to symmetry. The density matrix is expressed as

$$\rho_{ij}\approx \frac{1}{Z}\left[e^{-\beta H_{0}}-\beta \lambda e^{-\beta H_{0}}\sigma_{i}^{x}\sigma_{j}^{x}+\frac{{\left(\beta \lambda \right)}^{2}}{2}e^{-\beta H_{0}}\right],$$

with $e^{-\beta H_{0}}=\mathrm{diag}\left(p_{0}^{2},p_{0}p_{1},p_{0}p_{1},p_{1}^{2}\right)$ in the basis and

$$e^{-\beta H_{0}}\sigma_{i}^{x}\sigma_{j}^{x}=\left(\begin{array}{cccc} 0 & 0 & 0 & p_{0}^{2}\\ 0 & 0 & p_{0}p_{1} & 0\\ 0 & p_{0}p_{1} & 0 & 0\\ p_{1}^{2} & 0 & 0 & 0 \end{array}\right).$$

The reduced density matrix for patch *i* is obtained by tracing over *j*, $\rho_{i}\left(\lambda \right)={\mathrm{Tr}}_{j}\left(\rho_{ij}\right)$.

Because the trace over *j* of the first-order term in the perturbation vanishes, the correction to the reduced density matrix starts at second order. Expanding to this order, the probability of the ground state decreases as $p_{0}\left(\lambda \right)\approx p_{0}-0.043{\left(\beta \lambda \right)}^{2}$, while the excited state probability increases as $p_{1}\left(\lambda \right)\approx p_{1}+0.043{\left(\beta \lambda \right)}^{2}$, indicating that the occupation probability rises when the XX coupling is switched on.

Starting from the unperturbed eigenvalues $p_{0}$ and $p_{1}$, this shift in probabilities leads to the entropy shift

$$\delta S\approx 0.119{\left(\beta \lambda \right)}^{2}.$$

For instance, to achieve δS=0.007, we find λ≈0.010 given β≈25.13, which is consistent with the parameters used throughout this paper.

This analysis demonstrates that the weak XX correlation introduces a small but positive entropy increase of approximately 0.007 nat, which helps explain the elevation to 0.257 nat per patch observed in the full model. Such a modest adjustment underscores the sensitivity of the entropy to even tiny inter-patch interactions, reinforcing the robustness of our semi-classical framework.

## Appendix B. Proof That dS_hor_ + dS_rad_ ≥ 0

Throughout this appendix, we adopt the natural units defined in the Introduction for consistency and simplicity. For a Schwarzschild black hole characterised by mass *M*, the horizon area is given by $A=16\pi M^{2}$, and the corresponding Bekenstein–Hawking entropy is

$$S_{\mathrm{hor}}=\frac{A}{4}=4\pi M^{2}.$$

When the black hole loses energy dE=−dM through the emission of Hawking radiation, this induces a change in the horizon entropy, expressed as

(A1) $$dS_{\mathrm{hor}}=8\pi M\;dM=-\frac{dE}{T_{H}},\;T_{H}=\frac{1}{8\pi M}.$$

The Hawking radiation emitted by the black hole can be approximated as thermal radiation at temperature $T_{H}$. In line with Page’s coarse-grained prescription, we attribute to the emitted energy −dE an entropy contribution of

(A2) $$dS_{\mathrm{rad}}=\frac{4}{3}\;\frac{-dE}{T_{H}},$$

which holds for a relativistic bosonic gas in 3+1 dimensions. Here, the factor 4/3 represents the standard ratio of entropy density to energy density, s/ρ.

In our measurement-driven model, a subtle correction akin to a chemical potential arises because each outgoing quantum remains entangled with a distinct Planck-scale patch on the horizon. We denote this correction by δ, modifying the radiation entropy to

$$dS_{\mathrm{rad}}=\left(\frac{4}{3}+\delta \right)\frac{-dE}{T_{H}}.$$

As detailed in Appendix A, this correction is small, with $\delta ∼\lambda^{2}/\left(2\pi^{2}\right)\ll 1$. Combining the expressions in (A1) and (A2), the total change in entropy is

(A3) $$dS_{\mathrm{hor}}+dS_{\mathrm{rad}}=\left(-1+\frac{4}{3}+\delta \right)\frac{-dE}{T_{H}}=\left(\frac{1}{3}+\delta \right)\frac{-dE}{T_{H}}.$$

Since the black hole loses energy (dE<0) and the factor $\frac{1}{3}+\delta >0$, the overall product is non-negative:

$$dS_{\mathrm{hor}}+dS_{\mathrm{rad}}\;\geq \;0.$$

This inequality holds strictly unless dE=0, meaning no emission occurs. Consequently, the measurement process inherent to our model upholds the generalised second law, even as the horizon area diminishes due to evaporation.

This coarse-grained outcome, as captured in Equation (A3), parallels the initial phase of the Page curve, during which the entropy of the radiation increases more rapidly than the decrease in the Bekenstein–Hawking entropy of the horizon. At around the Page time, these two contributions reach equilibrium. Beyond this point, higher-order correlations among the patches—which are not accounted for in the present analysis—would need to be incorporated to observe the subsequent decline in $S_{\mathrm{rad}}$.

## Appendix C. Calculation of Average Entanglement Entropy

The average entanglement entropy $\langle S_{k}\rangle$ is given by

$$\langle S_{k}\rangle =\frac{15}{\pi^{4}}\int_{0}^{\infty }S_{k}\left(x_{k}\right)\cdot \frac{x_{k}^{3}}{e^{x_{k}}-1}\;dx_{k},$$

where $S_{k}\left(x_{k}\right)=\frac{x_{k}}{e^{x_{k}}-1}-\ln\left(1-e^{-x_{k}}\right)$. This integral can be evaluated analytically using series expansions to obtain an exact closed-form expression.

The integrand is defined as follows:

$$f\left(x_{k}\right)=S_{k}\left(x_{k}\right)\cdot \frac{x_{k}^{3}}{e^{x_{k}}-1}$$

For $x_{k}>0$, the terms are expanded using the following series:

$$\frac{1}{e^{x_{k}}-1}=\sum_{m=1}^{\infty }e^{-mx_{k}},\;-\ln\left(1-e^{-x_{k}}\right)=\sum_{r=1}^{\infty }\frac{e^{-rx_{k}}}{r}$$

The integral now becomes

$$\begin{array}{ccc} \int_{0}^{\infty }f\left(x_{k}\right)\;dx_{k} & = & \sum_{j,n\geq 1}\int_{0}^{\infty }x_{k}^{4}e^{-\left(j+n\right)x_{k}}\;dx_{k}\\ & + & \sum_{r,n\geq 1}\frac{1}{r}\int_{0}^{\infty }x_{k}^{3}e^{-\left(r+n\right)x_{k}}\;dx_{k} \end{array}$$

Using the standard Gamma-function integral $\int_{0}^{\infty }x^{p}e^{-ax}\;dx=\frac{\Gamma (p+1)}{a^{p+1}}$, the first term (p=4) is evaluated as

$$\int_{0}^{\infty }x_{k}^{4}e^{-\left(j+n\right)x_{k}}\;dx_{k}=\frac{\Gamma (5)}{{\left(j+n\right)}^{5}}$$

and the second term (p=3) as

$$\int_{0}^{\infty }x_{k}^{3}e^{-\left(r+n\right)x_{k}}\;dx_{k}=\frac{\Gamma (4)}{{\left(r+n\right)}^{4}}$$

yielding the following:

(A4) $$\int_{0}^{\infty }f\left(x_{k}\right)\;dx_{k}=24\sum_{j,n\geq 1}\frac{1}{{\left(j+n\right)}^{5}}+6\sum_{r,n\geq 1}\frac{1}{r}\frac{1}{{\left(r+n\right)}^{4}}$$

These sums are evaluated as follows: First,

$$\sum_{j,n\geq 1}\frac{1}{{\left(j+n\right)}^{5}}=\sum_{m=2}^{\infty }\frac{m-1}{m^{5}}$$

That is, for each m=j+n≥2, there are m−1 pairs (j,n) with j,n≥1, corresponding to j=1, m−1, and n=m−j. Using zeta functions, this becomes

$$\begin{array}{ccc} \sum_{m=2}^{\infty }\frac{m-1}{m^{5}} & = & \sum_{m=2}^{\infty }\left(\frac{1}{m^{4}}-\frac{1}{m^{5}}\right)\\ & = & \zeta (4)-\zeta (5) \end{array}$$

For the second sum,

$$\sum_{r,n\geq 1}\frac{1}{r}\frac{1}{{\left(r+n\right)}^{4}}=\sum_{r=1}^{\infty }\frac{1}{r}\sum_{m=r+1}^{\infty }\frac{1}{m^{4}}$$

For each fixed r≥1, let m=r+n. As *n* ranges from 1 to ∞, *m* ranges from r+1 to ∞, so $\sum_{n=1}^{\infty }\frac{1}{{\left(r+n\right)}^{4}}=\sum_{m=r+1}^{\infty }\frac{1}{m^{4}}$. The double sum is reordered as follows:

$$\sum_{m=2}^{\infty }\frac{1}{m^{4}}\sum_{r=1}^{m-1}\frac{1}{r}=\sum_{m=2}^{\infty }\frac{H_{m-1}}{m^{4}}$$

Given the relations $H_{m-1}=H_{m}-\frac{1}{m}$ and $\sum_{m=1}^{\infty }\frac{H_{m}}{m^{4}}=3\zeta \left(5\right)-\zeta \left(2\right)\zeta \left(3\right)$, it follows that

$$\sum_{m=2}^{\infty }\frac{H_{m}}{m^{4}}=\left(3\zeta \left(5\right)-\zeta \left(2\right)\zeta \left(3\right)\right)-1$$

Together with $\sum_{m=2}^{\infty }\frac{1}{m^{5}}=\zeta \left(5\right)-1$, this yields

$$\sum_{r,n\geq 1}\frac{1}{r}\frac{1}{{\left(r+n\right)}^{4}}=2\zeta \left(5\right)-\zeta \left(2\right)\zeta \left(3\right)$$

Combining these results gives

$$\int_{0}^{\infty }f\left(x_{k}\right)\;dx_{k}=24\zeta \left(4\right)-12\zeta \left(5\right)-6\zeta \left(2\right)\zeta \left(3\right)$$

and the average entanglement entropy is

$$\begin{array}{ccc} \langle S_{k}\rangle & = & 4-\frac{180\zeta (5)}{\pi^{4}}-\frac{15\zeta (3)}{\pi^{2}}\\ & \approx & 0.257\;\mathrm{nat} \end{array}$$

Numerical integration using a Riemann sum with a step size of Δx=0.0005 over $x_{k}\in \left[0,20\right]$ confirms $\langle S_{k}\rangle \approx 0.257$ nat, as the integrand $f(x_{k})$ stabilises beyond $x_{k}=20$ due to the exponential decay of $e^{-x_{k}}$, matching the analytical result within numerical precision.
